# Retinal oxygen extraction in individuals with type 1 diabetes with no or mild diabetic retinopathy

**DOI:** 10.1007/s00125-017-4309-0

**Published:** 2017-05-25

**Authors:** Klemens Fondi, Piotr A. Wozniak, Kinga Howorka, Ahmed M. Bata, Gerold C. Aschinger, Alina Popa-Cherecheanu, Katarzyna J. Witkowska, Anton Hommer, Doreen Schmidl, René M. Werkmeister, Gerhard Garhöfer, Leopold Schmetterer

**Affiliations:** 1 Department of Clinical Pharmacology, Medical University of Vienna, Währinger Gürtel 18-20, 1090 Vienna, Austria; 2 Department of Ophthalmology, Medical University of Warsaw, Warsaw, Poland; 3 Center for Medical Physics and Biomedical Engineering, Medical University of Vienna, Vienna, Austria; 4 Carol Davila University of Medicine and Pharmacy, Bucharest, Romania; 5 Department of Ophthalmology, Emergency University Hospital, Bucharest, Romania; 6 Department of Ophthalmology, Sanatorium Hera, Vienna, Austria; 7 Singapore Eye Research Institute, Singapore, Republic of Singapore; 8 Lee Kong Chian School of Medicine, Nanyang Technological University, Singapore, Republic of Singapore; 9 Imperial College, London, UK

**Keywords:** Diabetes mellitus, Diabetic retinopathy, Diabetic vascular diseases, Retinal blood vessels

## Abstract

**Aims/hypothesis:**

The aim of this study was to compare retinal oxygen extraction in individuals with diabetes with no or mild non-proliferative diabetic retinopathy and healthy age- and sex-matched volunteers.

**Methods:**

A total of 24 participants with type 1 diabetes and 24 healthy age- and sex-matched volunteers were included in this cross-sectional study. Retinal oxygen extraction was measured by combining total retinal blood flow measurements using a custom-built bi-directional Doppler optical coherence tomography system with measurements of oxygen saturation using spectroscopic reflectometry. Based on previously published mathematical modelling, the oxygen content in retinal vessels and total retinal oxygen extraction were calculated.

**Results:**

Total retinal blood flow was higher in diabetic participants (46.4 ± 7.4 μl/min) than in healthy volunteers (40.4 ± 5.3 μl/min, *p* = 0.002 between groups). Oxygen content in retinal arteries was comparable between the two groups, but oxygen content in retinal veins was higher in participants with diabetes (0.15 ± 0.02 ml O_2_/ml) compared with healthy control participants (0.13 ± 0.02 ml O_2_/ml, *p* < 0.001). As such, the arteriovenous oxygen difference and total retinal oxygen extraction were reduced in participants with diabetes compared with healthy volunteers (total retinal oxygen extraction 1.40 ± 0.44 vs 1.70 ± 0.47 μl O_2_/min, respectively, *p* = 0.03).

**Conclusions/interpretation:**

Our data indicate early retinal hypoxia in individuals with type 1 diabetes with no or mild diabetic retinopathy as compared with healthy control individuals. Further studies are required to fully understand the potential of the technique in risk stratification and treatment monitoring.

**Trial registration:**

ClinicalTrials.gov NCT01843114.

## Introduction

Diabetic retinopathy is a severe and sight-threatening complication of diabetes. The most important risk factors for diabetic retinopathy include duration of diabetes, poor glycaemic control and high BP [1]. During the development of diabetic retinopathy, a number of characteristic retinal abnormalities including microaneurysms, blot haemorrhages, cotton-wool spots and retinal venodilatation can be found, which are considered general signs of microvascular damage [2]. More than 60 years ago, it was recognised that hypoxia plays a role in the development of diabetic retinopathy [3]. The human retina receives its oxygen supply from two sources: the retinal and the choroidal vasculature [4]. The inner retina, including the retinal ganglion cells, is oxygenised via the retinal circulation, while the outer retina, including the photoreceptors, is oxygenised via the choroidal circulation.

No technique has previously, however, been available to measure oxygen extraction in the human retina. Various research groups have quantified hypoxia using different approaches in animal models of diabetes [5–10], but data are conflicting as to whether retinal hypoxia is present early in the disease process. Interpreting the results, one has to consider that many of the studies used microelectrode measurements that provide oxygen tension measurements directly in the tissue. This has the advantage that measurements are taken from well-defined locations, but has the disadvantage that no information is obtained on oxygen in the entire retina. Moreover, retinal neovascularisation, which is an important late-stage complication of human diabetic retinopathy, does not develop in any of the animal models studied [11]. A recent study in humans suggested reduced retinal oxygen extraction in individuals with type 2 diabetes based on measurements of retinal blood flow and retinal oxygen saturation, but retinal oxygen extraction was not quantified [12].

We have recently introduced a method for the measurement of total retinal oxygen extraction in humans [13]. This technique combines bi-directional Doppler optical coherence tomography (OCT) for measuring total retinal blood flow [14] with spectroscopic reflectometry for the measurement of oxygen saturation in retinal vessels [15]. Based on a mathematical model, total retinal oxygen extraction is calculated, representing the amount of oxygen that is extracted from the retinal circulation. In the present study, we hypothesised that changes in retinal oxygen extraction in individuals with type 1 diabetes with no or early signs of diabetic retinopathy would be detected with this technology. To verify this hypothesis, we performed a cross-sectional study comparing individuals with type 1 diabetes with age- and sex-matched healthy volunteers.

## Methods

### Participants

The study protocol was approved by the Ethics Committee of the Medical University of Vienna. The study was performed in adherence to the guidelines of the Declaration of Helsinki and Good Clinical Practice and all patients signed written informed consent. Twenty-four participants with type 1 diabetes with no or mild diabetic retinopathy and under good glycaemic control and 24 healthy volunteers were included in this cross-sectional study between January 2015 and August 2016. Diabetic retinopathy was graded using 7-standard field colour fundus photographs. All participants passed a screening examination, including a physical examination, BP measurement and ophthalmic examination. Exclusion criteria were age less than 18 years, ametropia of more than 6 dpt, best corrected visual acuity of less than 0.8, presence of any ocular pathologies, systemic hypertension (defined as systolic BP >145 mmHg or diastolic BP >90 mmHg, or a diagnosis of systemic hypertension in the medical history), clinically relevant illness prior to the study and pregnancy or lactation, as well as participation in a clinical study or intake of new medication in the 3 weeks before the study. Pupil dilation was achieved using one drop of 0.5% tropicamide (Mydriaticum ‘Agepha’, Agepha, Vienna, Austria). Measurements were taken after a resting period of 20 min to achieve stable haemodynamic conditions. In all participants, measurements were performed in the right eye.

### Measurement of retinal oxygen extraction

Measurement of retinal oxygen extraction was as described previously. Briefly, total retinal blood flow was measured using bi-directional Doppler OCT [14] and retinal vessel oxygen saturation was measured using spectroscopic reflectometry [15].

**Fig. 1:**
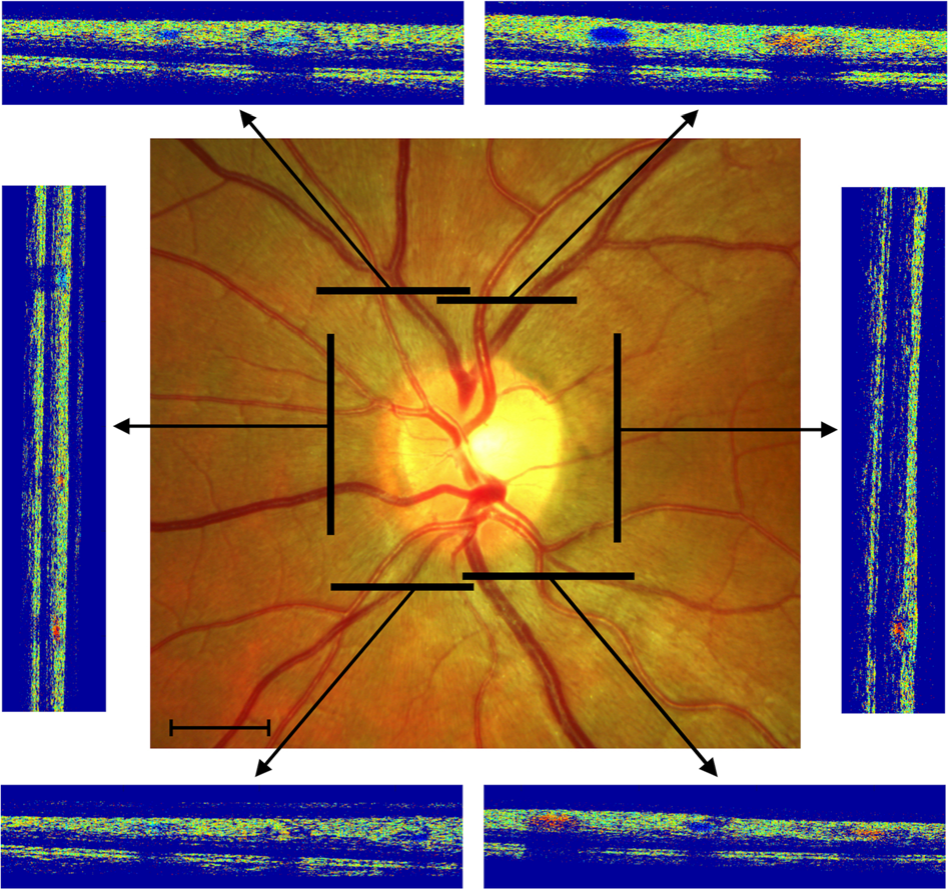
Sample measurement of total retinal blood flow and oxygen saturation. Black bars mark the measurement locations around the optic nerve head and the corresponding OCT images. The phase shifts, which result from the movements of blood in the vessels, are clearly visible in red or blue on the OCT images. Scale bar, 1000 μm

The bi-directional Doppler OCT is a custom-built system that allows the measurement of absolute blood velocity and absolute blood flow. Absolute blood velocity is quantified by measuring phase shifts of the complex OCT signals, and vessel cross-sectional areas are extracted from the phase images [16]. Measurements of retinal blood flow are taken from all arteries and veins above 30 μm in diameter around the optic nerve head, which includes virtually all blood entering and leaving the retinal circulation (Fig. 1). The measurement of blood flow in all retinal arteries and all retinal veins allows calculation of total arterial blood flow *Q*_*A,tot*_ and total venous blood flow *Q*_*V,tot*_ by summing up all arterial blood flow values *Q*_*A,i*_ and all venous blood flow values *Q*_*V,j*_ using the following respective equations:

$$ {Q}_{A, tot}=\sum_{i=1}^{nA}{Q}_{A, i} $$

$$ {Q}_{V, tot}=\sum_{j=1}^{nV}{Q}_{V, j} $$

Total retinal blood flow in the arteries and veins needs to be equal because the retina is an end organ. Hence, the total retinal blood flow *Q* is calculated as the mean between retinal arterial and venous blood flow:

$$ Q=\frac{Q_{A, tot}+{Q}_{V, tot}}{2} $$

Measurements were validated using in vitro experiments [17], and in vivo vs laser Doppler velocimetry [18] and invasive microsphere technology [19].

The OCT system is coupled to the commercially available Dynamic Vessel Analyzer (Imedos Systems, Jena, Germany), which includes an oxygen module that measures oxygen saturation in retinal arteries and veins based on fundus photographs [15]. Calculation of the retinal oxygen saturation is based on the fact that oxygenated and deoxygenated haemoglobin have different light-absorption characteristics. Two fundus photographs are taken at wavelengths of 610 and 545 nm, respectively. The first photograph taken is very close to the isosbestic point at 548 nm, at which oxygenated and deoxygenated haemoglobin show identical absorption. At a wavelength of 610 nm, oxygenated haemoglobin is nearly transparent. This allows for the calculation of oxygen saturation in retinal vessels. In the present study, oxygen saturation was measured in all retinal arteries (*SaO*_*2,A*_)_*i*_ and all retinal veins (*SaO*_*2,V*_)_*j*_. Previous studies indicate good reproducibility and validity of this technique [15, 20–23].

A mathematical model has been formulated to calculate total retinal oxygen extraction based on these measurements. This model takes into account the oxygen loss through the vascular wall between the entrance of the vessel at the optic disc and the point of measurement. In addition, it incorporates the facts that the oxygen saturation in different branch veins may differ and that blood merges into the central retinal vein. Finally, it is considered that part of the oxygen is not bound to haemoglobin, but is free, non-bound oxygen. From this, the oxygen content at the level of the central retinal artery (CRA) and the central retinal vein (CRV), i.e. *cO*_*2,CRA*_ and *cO*_*2,CRV*_, respectively, as well as the total retinal oxygen extraction *extO*_*2*_, can be calculated [13].

$$ \mathit{\operatorname{ext}}{O}_2=\left( c{O}_{2, CRA} - c{O}_{2, CRV}\right)\times Q $$

### Measurement of retinal thickness and retinal nerve fibre layer thickness

Structural OCTs were recorded using a commercially available spectral domain OCT system (Heidelberg Spectralis OCT, SPECTRALIS software version 5.3.3.0, EYE EXPLORER Software 1.6.4.0; Heidelberg Engineering, Heidelberg, Germany). To quantify retinal thickness, the average central thickness value from the SPECTRALIS thickness map report was taken. For the measurement of retinal nerve fibre layer thickness, the average thickness value from the SPECTRALIS RNFL analysis report was used.

### Measurement of BP and pulse rate

Systolic, diastolic and mean arterial BPs were measured on the upper arm using an automated oscillometric device (HP-CMS patient monitor; Hewlett Packard, Palo Alto, CA, USA). Pulse rate was automatically recorded by the same device from a finger pulse oximetric device.

### Statistical analysis

Data are presented as means ± SD. Differences between participants with diabetes compared with healthy control participants were studied using an unpaired *t* test. Bland–Altman plots were prepared to characterise the association between blood flow data as obtained in arteries and veins. A *p* value <0.05 was considered as the level of significance. Missing values were neither replaced nor estimated.

## Results

The main characteristics of the groups of healthy participants and those with diabetes are presented in Table 1. All participants with diabetes had either no (*n* = 22) or mild (*n* = 2) diabetic retinopathy. Mean disease duration in participants with diabetes was 16.2 ± 10.4 years. There were no significant differences in age, BP, intraocular pressure or pulse rate between the two groups. As expected, plasma levels of glucose and HbA_1c_ were higher in participants with diabetes than in healthy volunteers. No significant differences were found between the groups in terms of retinal thickness or retinal nerve fibre layer thickness.

**Table 1:** Characteristics of participants with diabetes and matched healthy volunteers

	Healthy volunteers (*n* = 24)	Participants with diabetes (*n* = 24)	*p* (between groups)
Age (years)	33 ± 11	33 ± 11	0.94
Sex (male/female)	7/17	7/17	1.00
HbA_1c_ (%)	5.2 ± 0.3	7.2 ± 1.6	<0.001
HbA_1c_ (mmol/mol)	33 ± 3.3	55 ± 17.5	<0.001
Blood glucose (mmol/l)	5.6 ± 0.8	9.8 ± 3.4	<0.001
Intraocular pressure (mmHg)	14 ± 2	14 ± 2	0.86
Systolic BP (mmHg)	119 ± 12	123 ± 12	0.21
Diastolic BP (mmHg)	75 ± 9	77 ± 9	0.63
Mean arterial pressure (mmHg)	93 ± 9	95 ± 9	0.39
Pulse rate (beats/min)	71 ± 10	75 ± 11	0.21
Total retinal blood flow in arteries (μl/min)	40.8 ± 5.5	46.2 ± 7.5	0.007
Total retinal blood flow in veins (μl/min)	39.9 ± 5.4	46.4 ± 7.5	0.001
Retinal thickness (μm)	231 ± 13	242 ± 24	0.15
Retinal nerve fibre layer thickness (μm)	97 ± 12	99 ± 8	0.62

Data are means ± SD, with the exception of sex

*p* < 0.05 was considered the level of significance

**Fig. 2:**
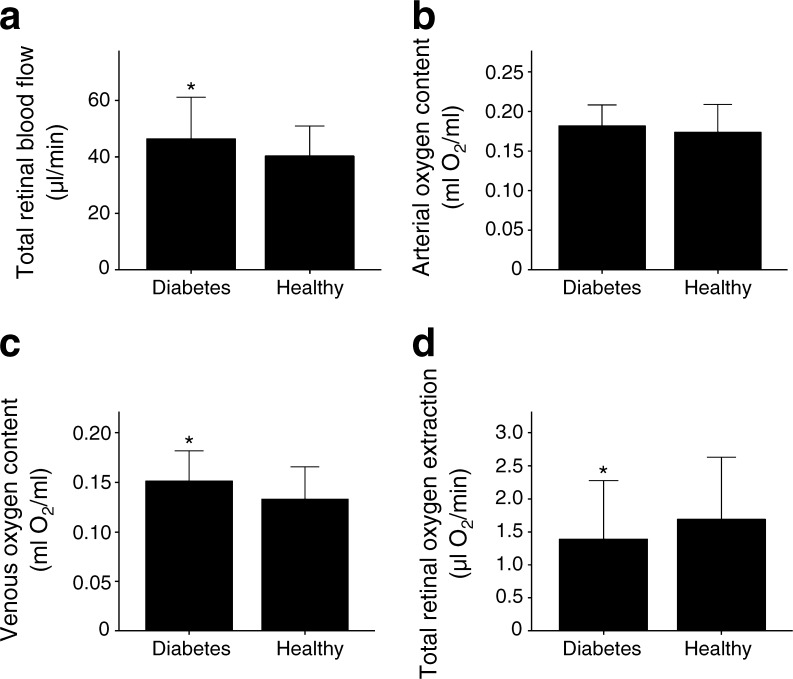
Total retinal blood flow (**a**), oxygen content in retinal arteries (**b**), oxygen content in retinal veins (**c**) and total retinal oxygen extraction (**d**) in participants with diabetes and healthy volunteers. Data are presented as means ± SD. *p < 0.05 between groups

**Fig. 3:**
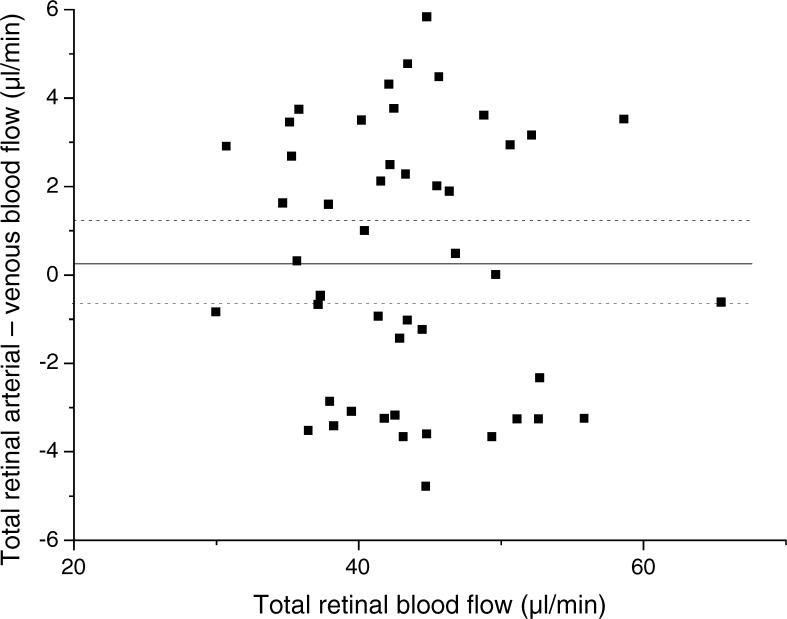
Bland–Altman plot showing differences between retinal blood flow as measured in arteries and retinal blood flow as measured in veins over the total retinal blood flow, as defined as the mean between values obtained in retinal arteries and veins. The solid line marks the mean difference between arteries and veins, while the dashed lines mark the mean difference ± 1.96 × SD. The mean difference was small (0.26 μl/min)

Total retinal blood flow was higher in participants with diabetes (46.4 ± 7.4 μl/min) than in healthy volunteers (40.4 ± 5.3 μl/min, *p* = 0.002 between groups, Fig. 2). This was also the case when total retinal blood flow values were compared in arteries or veins separately (Table 1). Differences between blood flow values in arteries and veins were small (Fig. 3), and no significant differences were seen when blood flow data were evaluated from either arteries or veins. Oxygen content in retinal arteries was comparable between the two groups (healthy control participants: 0.17 ± 0.02 ml O_2_/ml, participants with diabetes: 0.18 ± 0.01 ml O_2_/ml, *p* = 0.09; Fig. 2), but oxygen content in retinal veins was higher in participants with diabetes (0.15 ± 0.02 ml O_2_/ml) as compared with healthy volunteers (0.13 ± 0.02 ml O_2_/ml, *p* < 0.001; Fig. 2). As such, the arteriovenous oxygen difference was reduced in participants with diabetes vs healthy volunteers (0.030 ± 0.010 vs 0.041 ± 0.014 ml O_2_/ml, respectively). Calculating total retinal oxygen extraction revealed reduced values in participants with diabetes vs healthy volunteers (1.40 ± 0.44 vs 1.70 ± 0.47 μl O_2_/min, respectively, *p* = 0.03).

## Discussion

The present study shows reduced retinal oxygen extraction in individuals with type 1 diabetes with no or mild diabetic retinopathy, as compared with healthy individuals. Such changes were seen very early in the disease process when either no or minimal diabetic retinopathy was visible. This finding is compatible with early retinal hypoxia in diabetes. The most likely explanation is alterations in microvascular oxygen delivery, which would also explain the increase in retinal blood flow as a reaction to the insufficient oxygen supply of the retinal tissue. Another explanation could be retinal tissue damage resulting in lower oxygen consumption of the retinal tissue, but this seems unlikely given that no structural changes were observed in the present study using OCT. The technique used in the present study may have significant potential as a biomarker for diabetic retinopathy, and could be used for risk stratification and treatment monitoring.

As mentioned above, our technique of measuring retinal blood flow has been validated in several experiments. The excellent agreement between blood flow values obtained in arteries and veins in the present study is further proof of its reliability. Vessels smaller than 30 μm are not assessable with the current technique but, considering the small vessel diameters and the resulting low flow volumes of these vessels, the error introduced by this limitation can be estimated as less than 1% [14]. Total retinal blood flow has been quantified previously using a variety of different methods and the results are generally in the same range as reported in this paper [14, 24–26]. There is some controversy as to whether retinal blood flow is increased [27, 28] or decreased [12, 29–31] in diabetes. Results may depend on the stage of diabetic retinopathy, type and duration of diabetes, presence of comorbidities and use of concomitant medication [28]. Our results are, however, in good agreement with our previous study in a comparable group of individuals with type 1 diabetes [32].

A variety of previous studies have reported on oxygen saturation in the retinal vessels of individuals with diabetes [12, 33–36]. In keeping with the present study, increased oxygen saturation in retinal arteries and veins and decreased retinal arteriovenous oxygen difference have been reported. Without measurement of retinal blood flow it is, however, difficult to interpret these results in terms of retinal metabolism or retinal oxygenation.

There is a long-standing debate as to whether neurodegenerative damage or microvascular alterations occur earlier in individuals with diabetes [37, 38]. In the present study, reduced retinal oxygen extraction was detected at a stage when the retinal nerve fibre layer and retinal thickness, as measured with standard OCT, were not significantly different between the diabetes group and the healthy control group. However, it is important to take into account the consideration that this question is difficult to answer from a clinical study, because which change can be detected earlier depends to a large degree on the performance characteristics of the investigatory method employed. Moreover, longitudinal studies are required for collecting repeated outcome measures, because of the considerable interindividual variability of functional and structural measurements in humans.

The present study has several limitations that need to be mentioned. The sample size was small and only individuals with type 1 diabetes were included. The degree to which our results can be extrapolated to individuals with type 2 diabetes is uncertain. In type 2 diabetes, retinal oxygen extraction may be affected by factors such as older age, hypertension or hyperlipidaemia. In addition, we focused on a group of individuals with relatively early diabetes and good glycaemic control in order to reduce the influence of any potentially required systemic medications on our results. We cannot exclude, however, that glucose plasma levels influenced our results by inducing retinal vasodilatation [39, 40]. Nevertheless, we have previously shown that blood flow decreases during euglycaemic insulin clamps [32]. This may indicate that retinal oxygen extraction would decrease as well, although further studies are required to support this hypothesis.

The current method for measuring retinal oxygen extraction is non-invasive and non-contact, and showed good feasibility in this group of individuals. This technique can, in principle, also be employed for large cohorts, with the limitation of sufficient visual fixation. As of now, however, the technique is not commercially available. Although both methods—measurement of total retinal blood flow and measurement of oxygen saturation—have been previously validated, as mentioned above, it is difficult to validate retinal oxygen extraction values because of the lack of any reference method. We have, however, previously shown that during 100% oxygen breathing, retinal oxygen extraction is largely reduced as expected from previous animal experiments [41]. Total retinal oxygen extraction levels were slightly lower than those obtained in our previous study [13], which included very few and younger participants compared with the present study. With currently existing technology, it is not possible to assess local oxygen extraction or tissue oxygen levels in vivo. Our results are limited to measurements of larger retinal vessels with a diameter of 30 μm or more, because neither oxygen saturation nor blood flow can be quantified in smaller vessels. Finally, we cannot exclude that alterations in oxygenation of the retina resulted from choroid changes during the course of diabetic retinopathy, thereby counteracting the oxygen deficit from the retina. There are, however, no data in the literature to support this hypothesis.

In conclusion, the present study indicates that individuals with early type 1 diabetes have reduced retinal oxygen extraction. The technique used in this study may have considerable potential in quantifying the degree of hypoxia in the diabetic retina. Further studies in larger cohorts are required to gain more insight into retinal oxygenation at different stages of diabetic retinopathy.

## Abbreviations

CRA: Central retinal artery
CRV: Central retinal vein
OCT: Optical coherence tomography
